# The Utility of Perirenal Fat in Determining the Risk of Onset and Progression of Diabetic Kidney Disease

**DOI:** 10.1155/2022/2550744

**Published:** 2022-11-30

**Authors:** Hongtu Hu, Wei Liang, Zongwei Zhang, Zikang Liu, Fan Chu, Yan Bao, Jialu Ran, Guohua Ding

**Affiliations:** ^1^Division of Nephrology, Renmin Hospital of Wuhan University, 238 Jiefang Rd, Wuhan, Hubei 430060, China; ^2^Key Clinical Research Center of Kidney Disease, 238 Jiefang Rd, Wuhan, Hubei 430060, China; ^3^Division of Endocrinology, Renmin Hospital of Wuhan University, 238 Jiefang Rd, Wuhan, Hubei 430060, China; ^4^Department of Biostatistics and Bioinformatics, Rollins School of Public Health, Emory University, 1518 Clifton Rd, Atlanta, Georgia 30322, USA

## Abstract

**Background:**

Perirenal fat (PRF) has multiple effects on the kidney through its physical structure and adipocytokine-secreting ability. The present study explored the relationship between PRF thickness and the onset and progression of albuminuria in patients with diabetes.

**Methods:**

In the cross-sectional analysis, we screened 959 patients from 8764 subjects with type 2 diabetes mellitus (T2DM) who met the inclusion criteria and measured their perirenal fat thickness (PFT) using color Doppler ultrasound. A group of laboratory indexes were included in the analysis models. In a longitudinal study, a total of 218 patients with a baseline UACR <30 mg/g were included in the follow-up study.

**Results:**

In a cross-sectional analysis, patients with diabetes and higher PFT presented with higher albuminuria. Multiple logistic regression analysis indicated that PFT was an independent risk factor for the degree of albuminuria in patients with T2DM (odds ratio = 4.186, 95%CI: 2.290–7.653, *P* < 0.001). In a longitudinal study, 218 albuminuria-free patients with T2DM at the baseline were followed up for a mean of 12.3 months. Based on the cutoff value from the ROC diagnostic test in the cross-sectional study, patients were divided into two groups: higher PFT (H-PFT) and lower PFT (L-PFT). Kaplan–Meier survival curve analysis showed that H-PFT was associated with a higher incidence of albuminuria than L-PFT (log-rank test, *χ*2 = 4.522, *P* = 0.033). Cox regression analysis showed that PFT was a risk factor for the earlier onset of albuminuria (hazard ratio 2.83, 95% CI: 1.34–4.88, *P* < 0.001).

**Conclusions:**

PRF evaluated by color Doppler ultrasound is an easy and reliable tool for predicting the onset and progression of albuminuria in patients with T2DM.

## 1. Introduction

Diabetic kidney disease (DKD) is the most frequent and severe complication of diabetes mellitus (DM). Approximately 40% of patients with diabetes present with proteinuria and renal dysfunction and DKD reportedly account for 50% of newly diagnosed end-stage renal disease (ESRD) [1, 2]. Metabolic disorders caused by hyperglycemia in DKD can lead to both glucose and lipid metabolism disorders. Adipose tissue, an organ that stores energy and releases endocrine factors, participates in various physiological processes by secreting adipokines and cytokines, thereby maintaining lipid metabolic homeostasis and energy balance [3]. Adipose tissue consists of visceral adipose tissue (VAT) and subcutaneous adipose tissue (SAT) [4]. Accumulating evidence suggests that the abnormal distribution and ectopic accumulation of VAT in humans are more closely associated with cardiovascular risk factors, such as insulin resistance, dyslipidemia, hypertension, and atherosclerosis [5].

Perirenal fat (PRF) is a subtype of VAT located in the retroperitoneal space encapsulating the kidneys and filling the space between the kidney and the adjacent retroperitoneal tissue [6]. PRF has been reported to be associated with renal disease in various ways [7, 8]. Previous studies showed that PRF was associated with a loss in renal function in patients with diabetes [9–11]. In addition, PRF was positively associated with the degree of albuminuria in a cross-sectional study with a small sample size of patients with type 2 diabetes mellitus (T2DM) [12]. However, the value of PRF in predicting the onset of albuminuria in patients with diabetes warrants further longitudinal studies.

The present study investigated the relationship between PRF and albuminuria in a large sample of patients with T2DM at both cross-sectional and longitudinal levels. A threshold value for perirenal fat thickness (PFT) in patients with diabetes was set to render a noninvasive and reliable indicator for predicting the occurrence of early stage DKD.

## 2. Materials and Methods

### 2.1. Study Participants

Patients with T2DM aged 18–70 were included in this study. T2DM was diagnosed according to American Diabetes Association (ADA) Diabetes Diagnostic Criteria [13]. The exclusion criteria were (1) patients with incomplete clinical data, (2) patients without renal ultrasound records, (3) patients with a history of malignancy and surgical trauma, and (4) patients diagnosed with nondiabetic renal diseases. Based on the inclusion and exclusion criteria, 959 patients with T2DM treated at the Renmin Hospital of Wuhan University from December 2019 to April 2021 were enrolled in this cross-sectional study. Based on the degree of the urine albumin-creatinine ratio (UACR) [14], patients with diabetes were divided into three groups: subjects without albuminuria (DM-1 group, UACR <30 mg/g, 636 cases), subjects with microalbuminuria (DM-2 group, 30 mg/g < UACR < 300 mg/g, 191 cases), and subjects with macroalbuminuria (DM-3 group, UACR > 300 mg/g, 132cases). Moreover, 100 healthy individuals who signed informed consent forms for clinical research from the health examination center in our hospital were included in the control group (CG group).

**Figure 1:**
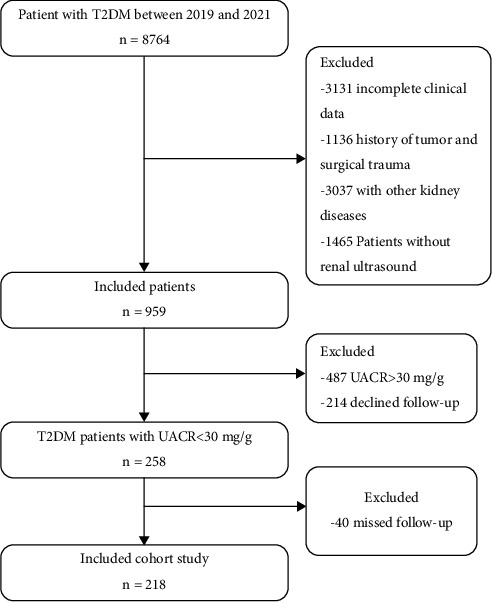
Flow chart of the study.

Additionally, patients with a baseline UACR < 30 mg/g were recruited for the longitudinal study. A total of 218 patients with confirmed consent forms were followed up for a median time of 12.3 months (range: 9.4, 13.1). The composite endpoints for renal outcomes were UACR >30 mg/g and total 24-hour urinary protein (UTP) > 0.15 g/24 h. Based on the cutoff value from the receiver operating characteristic (ROC) diagnostic test in the cross-sectional study and according to their median PFT, the subjects in the longitudinal study were assigned to the group with increased PFT (H-PFT group) and the group with decreased PFT (L-PFT group) at the baseline. A flow chart of the included human subjects is presented in Figure 1. The study was approved by the ethics committee of Renmin Hospital of Wuhan University to access the digital records of subjects who signed informed consent forms to share digital records with local institutions (WDRY2021-KS034). This study was approved by the China Clinical Trial Registry (ChiCTR2100052518, https://www.chictr.org.cn).

### 2.2. Data Collection

Demographic indexes, such as sex, age, weight, and height, were accessed from the digital records. Body mass index (BMI) was defined as the ratio of the weight (kg) to the square of the height (m^2^). Fasting venous blood was collected to measure hemoglobin (Hb), white blood cell (WBC) count, red blood cell (RBC) count, platelet (PLT) count, urea nitrogen (urea), blood creatinine (SCr), blood uric acid (UA), total cholesterol (TCh), total triglycerides (TG), high-density lipoprotein cholesterol (HDL-Ch), low-density lipoprotein cholesterol (LDL-Ch), and fasting plasma glucose (FPG) levels. The predicted glomerular filtration rate (eGFR) was calculated using the simplified modified diet trial for renal disease (MDRD) formula: eGFR (ml/min/1.73 m^2^) = 175 × (SCr (mg/dl))^−1.234^ × (age)^−0.179^ × (0.79 (female)). The UACR was calculated with the formula: UACR (mg/g) = urinary microalbumin/urinary creatinine. The three ranges of UACR (<30 mg/g, 30 mg/g ≤ UACR ≤ 300 mg/g, and >300 mg/g) were defined as no albuminuria, microalbuminuria, and macroalbuminuria, respectively [14].

### 2.3. Ultrasound Measurement of PFT

**Figure 2:**
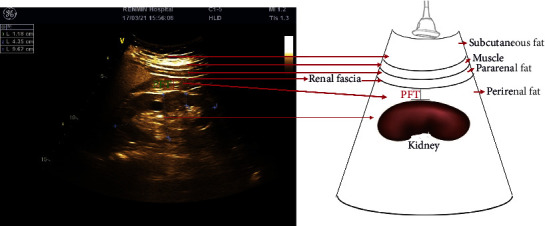
Schematic diagram of PFT measurement.

Ultrasonography of the kidneys from individual subjects was performed using a duplex Doppler instrument (Acuson Sequoia 512 ultrasound system, Siemens, USA). PFT was measured according to a previously described protocol [11]. Briefly, PRF was the thickness of the fat capsule along the long axis of the kidney, and pararenal fat was the thickness of the fat layer measured between the medial surface of abdominal muscle tissue and the renal fascia. PRF was measured while the patient was in the supine position. The probe was held vertical to the skin on the transverse aspects of the abdomen and slowly moved laterally until the optimal position was found. Longitudinal scanning was performed with the kidney surface almost parallel to the skin. The pressure on the probe should be as small as possible to prevent the fat layer from being compressed. The thickness of the fat capsule was quantified by the vertical distance between the renal fascia and the surface of the kidney, also defined as the PFT, which was measured from both sides of the kidney and averaged from individual subjects by three experienced technologists with blind group information. The correlation between PFT values measured on both sides was 0.45 (*P* < 0.001) (Figure S1). The interoperator agreement between the three experienced technologists is 0.89. The schematic diagram of PFT measurement is presented in Figure 2.

### 2.4. Statistical Analysis

Origin (version 2021b; Origin Lab, Inc., Northampton, MA, USA) was used for graphing the data, and data analysis was performed using SPSS (version 22; SPSS, Inc., Chicago, IL, USA). Data were expressed as means ± SD or as medians with quartiles according to the distribution characteristics. *T*-test, the Mann–Whitney *U* test, and one-way analysis of variance (ANOVA), and Tukey multiple comparison test were used to compare the differences between two or more groups. Logistic regression analysis was performed for the univariate analysis, which included variables with a significant difference. Kaplan–Meier survival analysis and log-rank tests were performed to compare the incidence of albuminuria onset in subjects with different PFTs. Cox univariate and multifactor regression analyses were performed to estimate the risk factors for albuminuria onset and progression to diabetes. Statistical significance was set at *P* < 0.05.

## 3. Results

### 3.1. Cross-Sectional Analysis in Patients with Diabetes

A total of 959 patients were enrolled in this cross-sectional analysis. The clinical characteristics of patients with diabetes (DM group) and healthy subjects without diabetes (CG group) are presented in Table 1. The HbA1C, FPG, UA, and PFT thickness levels were higher in the DM group than in the CG group, whereas the HDL and ALB levels were lower in the DM group than in the CG group (*P* < 0.05).

Among the subjects with diabetes, the DM-1, DM-2, and DM-3 groups were divided according to the degree of albuminuria. A significant difference was observed in the PFT, HbA1C, UTP, age, urine creatinine, urine microalbumin, UACR, ALB, urea, SCr, UA, TG, LDL, eGFR, Hb, PT, duration of diabetes mellitus, and hypertension levels among the three DM subgroups (*P* < 0.05) (Table 2). An increasing trend from the DM-1 group to the DM-3 group was indicated in age, duration of diabetes mellitus, UTP, UACR, urea, SCr, UA, PFT, and PFT/BMI.

**Figure 3:**
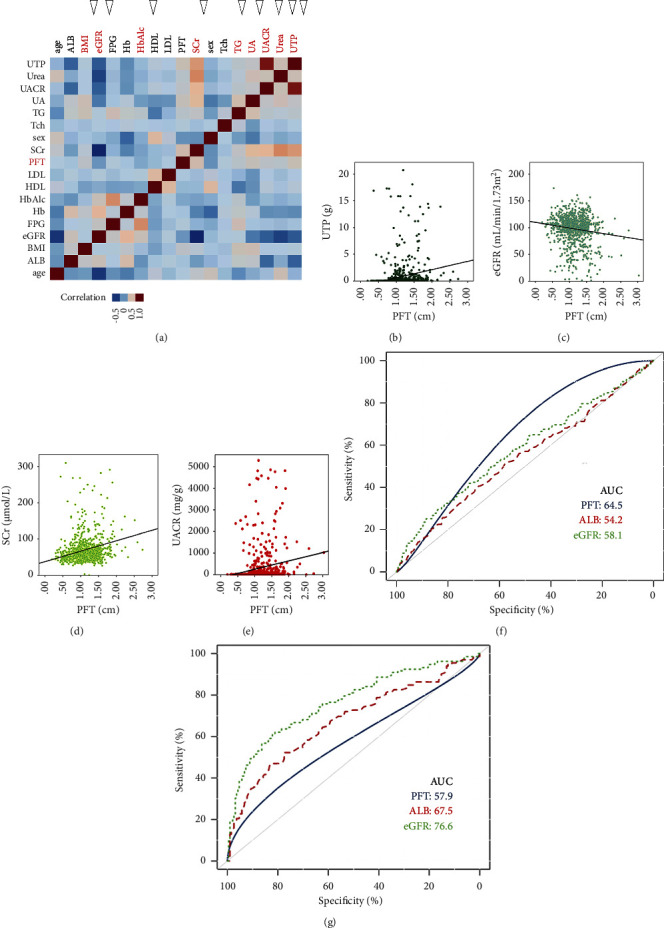
Correlation analysis and ROC analysis. (a) Heat map of correlation of clinical indicators in patients. Values with arrows are related to PFT (*P* < 0.05). (b) Scatter plot between total 24-hour urinary protein (UTP) and perirenal fat thickness (PFT). (c) Scatter plot between predicted glomerular filtration rate (eGFR) and PFT. (d) Relationship between blood creatinine (SCr) and PFT. (e) Scatter plot between urinary creatinine protein ratio (UACR) and PFT. (f) ROC diagnostic test for the progression of diabetes without proteinuria to diabetes with microproteinuria. (g) ROC diagnostic test for the progression of diabetes with microproteinuria to diabetes with significant proteinuria.

According to the Pearson correlation analysis, PFT thickness was positively correlated with the degree of UTP, UACR, urea, SCr, UA, TG, BMI, and the history of hypertension (Table S1). In contrast, a negative correlation (Figure 3) was found between PFT, HbA1C, and eGFR (*P* < 0.05). The heatmap in Figure 3(a) describes the mutual relationship between the two indexes with significant differences among the subgroups of DM. Representative distribution patterns with PFT are presented in Figures 3(b)–3(e).

To compare the ability to predict the different stages of DKD, the parameters of ALB, eGFR, and PFT were included in the ROC curve. As shown in Figure 3(f), PFT can better predict the onset of albuminuria (damaging albuminuria to microalbuminuria). In contrast, the power of PFT to predict the stage of microalbuminuria to macroalbuminuria was lower than that of ALB and eGFR (Figure 3(g)). Moreover, the cutoff value [1] of PFT for predicting a new onset of albuminuria was 0.90 cm (sensitivity, 96.3%; specificity, 30.5%; and Youden index, 1.27). The cutoff value [2] of PFT for predicting the (Figure 2) worsening of albuminuria was 1.56 cm (sensitivity, 28.0%; specificity, 88.48%; and Youden index, 1.17).

Using proteinuria as the endpoint, multiple logistic regression showed that increased UTP, increased PFT, increased TG, and age were the risk factors for the onset of proteinuria (*P* < 0.05), as shown in Table S2, and a scoring system for risk factors for the presence of proteinuria was obtained (Figure S2A). When the presence of proteinuria was used as the endpoint time, multiple logistic regression showed that increased BMI, male sex, and increased UACR were risk factors for thickened PFT (*P* < 0.05), as shown in Table S3, and a scoring system for risk factors for increased PFT was obtained (Figure S2B).

### 3.2. Follow-Up Analysis of Patients with Albuminuria-Negative Diabetes

**Figure 4:**
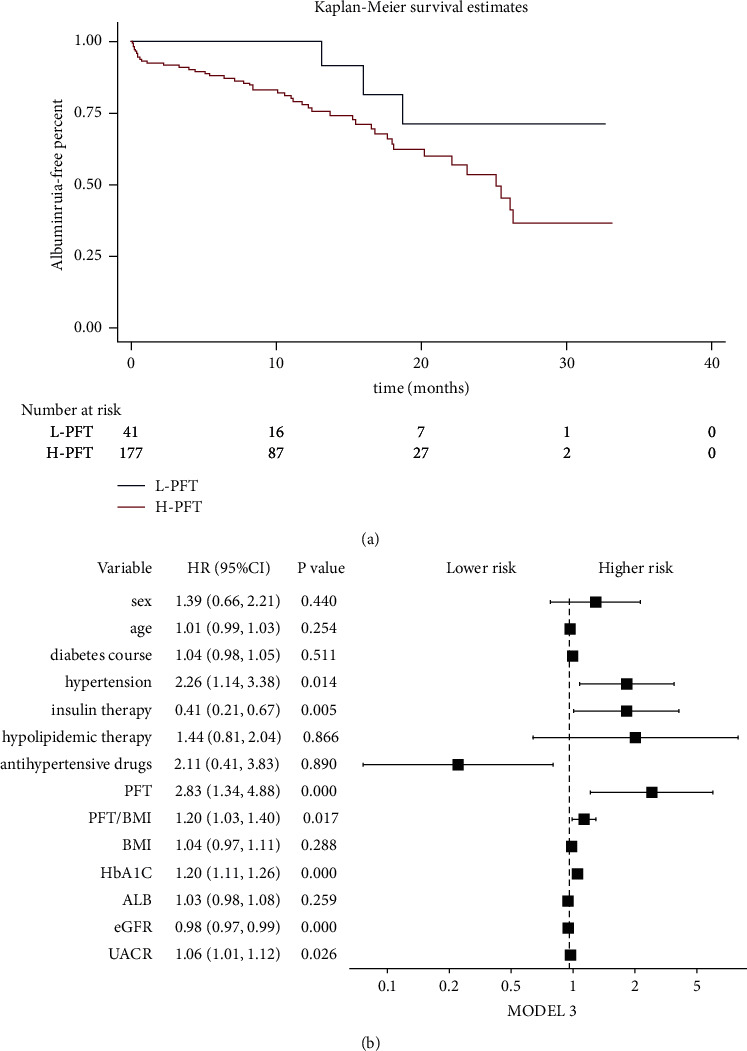
KM survival curve plot and COX model forest plot. (a) Renal survival in the L-PFT group versus the H-PFT group. (b) COX model 3 forest plot (demographic parameters + history of disease + concomitant medications + clinical indexes + PFT).

Based on the cutoff value from the ROC diagnostic test in the cross-sectional part, 218 DM patients without albuminuria enrolled in the follow-up study were divided into two groups: L-PFT (*n* = 41) and H-PFT (*n* = 177). The baseline characteristics of all the subjects in the longitudinal study are shown in Table 3. Albuminuria was set as the endpoint for a median follow-up time of 12.3 months; three patients (7.3%) with L-PFT reached this endpoint, including two patients with UACR > 30 mg/g and one with UACR > 300 mg/g. In contrast, 46 patients (26.0%) with H-PFT reached the endpoint, which included 32 with UACR > 30 mg/g and 14 with UACR > 300 mg/g. A Kaplan–Meier survival curve indicated a significantly higher incidence in the H-PFT group than in the L-PFT group (log-rank test, *χ*2 = 4.522, *P* = 0.033), as shown in Figure 4(a).

Multivariate Cox regression models were used to estimate the risk factors for albuminuria onset. Various models (model 1: demographic parameters + PFT; model 2: demographic parameters + history of disease + concomitant medications + PFT; and model 3: demographic parameters + history of disease + concomitant medications + clinical indexes + PFT) were tested, and PFT was found to be an independent risk factor for the onset of albuminuria in patients with albuminuria-negative DM (model 1: HR 2.91, 95% CI: 1.16–7.30, *P* = 0.003; model 2: HR 2.89, 95% CI: 1.14–7.50, *P* = 0.026; and model 3: HR 2.83, 95% CI: 1.34–4.88, *P* < 0.001), as shown in Table 4. Forest plots of the individual models are shown in Figure 4(b).

## 4. Discussion

This study found that PRF was thickened in patients with diabetes. Additionally, PRF thickness measured by ultrasound was associated with albuminuria in different stages of DKD in cross-sectional and longitudinal studies, rendering it an easy and practical approach to predict the onset and progression of albuminuria in patients newly diagnosed with diabetes.

Traditionally, PRF has been considered a specific component of visceral fat. An increasing numbers of studies have reported that PRF is associated with the progression of various chronic diseases, such as cardiovascular disease, DM, and renal disease [15]. Our study showed that PFT levels were higher in patients with diabetes than in healthy controls. In addition, remarkable PRF was also present in diabetic subjects with albuminuria compared to those without albuminuria. After adjustment for confounders, logistic regression analysis showed that increased PFT was an independent risk factor for the development of proteinuria in patients with diabetes. In the longitudinal study, patients with thicker PRF had a poorer prognosis and were more likely to develop proteinuria. Therefore, a close relationship between proteinuria in patients with DKD and thickened PFT has been proposed. A previous study reported the relationship between perirenal fat and proteinuria in DKD using a small sample size [12]. Additionally, a recent study [9] applied computerized tomography (CT) to outline PFT in diabetic patients and indicated that PFT was a more sensitive tool for monitoring renal function loss than other VAT. Compared to the precise measurement of the PFT by CT, ultrasound is less accurate. However, B-mode ultrasound imaging is easy to access and manipulate to evaluate kidney morphology and PFT parameters. The present study indicates that a PFT cutoff value of 0.90 cm is reliable for predicting the onset of albuminuria in subjects with T2DM. Moreover, the cutoff value of 1.56 cm for PFT is useful for predicting the progression of DKD in patients. Both BMI and age are well-accepted risk factors for onset of DKD [16]. Intriguingly, the thickness of PRF was also associated with the state of DKD independent with age and BMI in the present study, but whether PRF is a dependent marker of kidney function disorder in a diabetic environment needs further studies.

Dyslipidemia is a metabolic disorder in patients with T2DM that contributes to worsening renal function and proteinuria in DKD [17, 18]. In our study, TG and PFT levels were independent risk factors for proteinuria in DKD. However, TG and other serum lipid parameters, such as Tch and LDL, had weak relationships with the thickness of PRF, suggesting that the source of PRF is independent of systemic lipid metabolism in the diabetic environment.

Renal lipid accumulation toxicity (lipotoxicity) is believed to promote kidney disease progression [19, 20]. It has been found that patients with DKD have increased cholesterol synthesis and impaired efflux from renal cells, increased fatty acid uptake, and decreased fatty acid oxidation [21]. Several studies have reported an association between increased visceral fat, remarkably increased PRF, metabolic and cardiovascular disease determinants, and renal dysfunction. The mechanisms by which PRF affects renal injury have not been fully elucidated.

It has been suggested that PRF may influence the development and progression of renal disease by affecting the RAS system and secretion of multiple adipokines and inflammatory cytokines [22]. The proximal location of PRF in the kidney makes its specific anatomical and morphological features closely related to the development and progression of renal disease. Despite its small volume compared to the subcutaneous fat and other sites of visceral fat deposition, PRF plays an essential role in the maintenance and homeostasis of renal function through various mechanisms. In addition, PRF is considered a reservoir of mesenchymal stem cells that can differentiate into adipocytes, osteoblasts, chondrocytes, and epithelial cell lineages [23]. However, little is known about the origin of the perirenal adipocytes. In DKD, the origin of PRF thickening needs to be investigated in depth.

The present study had some limitations as follows: The sample size of the longitudinal study was relatively small. Since albuminuria is a time-dependent complication of diabetes, a longer follow-up time could strengthen the predictive value of PRF for the onset of albuminuria. Population recruitment from multiple centers in different ethnic regions could reduce the selection bias. However, the specific mechanism of PRF thickening and its effect on renal function have not yet been elucidated.

## 5. Conclusion

Thickened PRF is a clinical feature of albuminuria-positive patients with T2DM and is an independent risk factor for the development of proteinuria in DKD. Based on the potential functions of PRF, accumulation of PRF is an alternative therapeutic target to prevent the onset and progression of DKD.

## Figures and Tables

**Table 1:** Comparison of clinical characteristics between the CG group and DM group.

	CG (*n* = 100)	DM (*n* = 959)	*t*	*P*
Gender (M/F)	47/53	609/350	−0.143	0.457
Age (years)	59 (51–68)	60 (49–70)	−1.211	0.110
HbA1C (%)	5.87 ± 1.34	9.49 ± 2.58	−22.655	**<0.001**
FPG (mmol/L)	5.47 ± 1.92	8.89 ± 3.80	−14.995	**<0.001**
ALB (g/L)	47.64 ± 3.05	40.61 ± 5.30	20.087	**<0.001**
Urea (mmol/L)	6.19 ± 1.68	6.18 ± 3.58	0.022	0.983
UA (*μ*mol/L)	336.70 ± 112.62	359.40 ± 108.84	−1.978	**0.048**
Tch (mmol/L)	4.98 ± 1.06	5.12 ± 12.24	−0.117	0.907
TG (mmol/L)	2.13 ± 2.29	2.17 ± 2.05	−0.178	0.859
HDL (mmol/L)	1.24 ± 0.38	1.03 ± 0.32	6.164	**<0.001**
LDL (mmol/L)	3.07 ± 0.98	2.93 ± 5.75	0.225	0.882
Rx				
None (diet alone)	—	86 (9.0)	—	—
OHA (*n*, %)	—	236 (24.6)	—	—
Insulin ± OHA (*n*, %)	—	568 (59.2)	—	—
Antihypertensive drugs (*n*, %)	—	430 (44.8)	—	—
ACE-I/ARBs (*n*, %)	—	252 (26.3)	—	—
Hypolipidemic therapy (*n*, %)	—	324 (33.8)	—	—
PFT (cm)	0.85 (0.62–1.09)	1.20 (0.92–1.38)	−12.442	**<0.001**
BMI (kg/m^2^)	20.43 ± 1.11	24.66 ± 3.98	−8.465	**<0.001**
PFT/BMI (mm/kg/m^2^)	0.41 (0.32, 0.50)	0.52 (0.40, 0.61)	−13.233	**<0.001**

CG: health control group; DM: type 2 diabetes group; HbA1C: glycated hemoglobin ratio; FPG: fasting plasma glucose; ALB: serum albumin; urea: serum urea nitrogen; UA: blood uric acid; Tch: total serum cholesterol; TG: triglycerides; HDL: serum high-density lipoprotein; LDL: serum low-density lipoprotein; PFT: perirenal fat thickness; BMI = weight (kg)/height^2^ (m^2^); OHA: oral hypoglycemic agent; Rx, prescription.

**Table 2:** Comparison of clinical characteristics among the DM-1 group, DM-2 group, and DM-3 group.

	DM-1 (*n* = 636)	DM-2 (*n* = 191)	DM-3 (*n* = 132)	*P*
Gender (M/F)	397/239	119/72	93/39	0.203
Age (years)	57 (48–65)	60 (51–69)	61 (51–70)	**<0.001**
Diabetes course (years)	5 (1–10)	8 (1–10)	10 (5–14)	**<0.001**
Hypertension course (years)	0 (0–5)	0 (0–10)	5 (1–10)	**<0.001**
Body weight	68.12 ± 12.97	68.53 ± 14.21	69.10 ± 12.96	0.722
Height (m)	1.66 ± 0.08	1.66 ± 0.78	1.66 ± 0.72	0.545
BMI (kg/m^2^)	24.6 ± 3.9	24.9 ± 4.0	24.7 ± 4.0	0.545
HbA1C (%)	9.48 ± 2.73	9.83 ± 2.18	8.95 ± 4.20	**0.025**
FPG (mmol/L)	8.78 ± 3.85	9.18 ± 3.40	8.94 ± 4.25	**<0.001**
UTP (g/24 h)	0.06 ± 0.67	0.28 ± 0.21	5.28 ± 6.31	**<0.001**
UCr (*μ*mol/L)	9458 ± 5177	7466 ± 4241	5786 ± 3571	**<0.001**
ALB (g/L)	41.28 ± 4.77	40.57 ± 5.56	37.57 ± 6.19	**<0.001**
Urea (mmol/L)	5.68 ± 3.42	6.19 ± 2.29	8.84 ± 4.82	**<0.001**
SCr (*μ*mol/L)	59.8 ± 16.7	72.6 ± 54.8	123.1 ± 87.2	**<0.001**
UA (*μ*mol/L)	348.5 ± 105.3	363.9 ± 104.8	406.7 ± 116.5	**<0.001**
Tch (mmol/L)	1.96 ± 1.65	5.83 ± 16.50	4.88 ± 1.34	0.676
TG (mmol/L)	1.96 ± 1.65	2.56 ± 2.82	2.56 ± 2.23	**<0.001**
HDL (mmol/L)	1.02 ± 0.31	1.02 ± 0.29	1.09 ± 0.38	0.086
LDL (mmol/L)	2.72 ± 1.76	2.57 ± 1.16	4.58 ± 15.19	**0.002**
eGFR (mL/min/1.73 m^2^)	104.2 ± 19.4	97.8 ± 24.2	67.9 ± 33.0	**<0.001**
Hb (mg/L)	137.1 ± 21.4	135.2 ± 22.1	123.4 ± 23.4	**<0.001**
PT (s)	10.70 ± 0.83	10.90 ± 1.48	10.67 ± 0.90	**0.030**
Rx, *n* (%)				
None (diet alone)	53 (8.3)	22 (11.8)	11 (8.0)	0.251
OHA	128 (20.1)	67 (35.1)	41 (31.4)	**0.024**
Insulin ± OHA	397 (62.4)	91 (47.7)	80 (60.5)	0.141
Antihypertensive drugs	250 (39.3)	97 (50.9)	83 (63.2)	**<0.001**
ACE-I/ARBs	134 (21.1)	58 (30.4)	60 (45.1)	**<0.001**
Hypolipidemic therapy	198 (31.1)	73 (38.4)	53 (40.2)	0.251
PFT (cm)	1.08 (0.85–1.33)	1.15 (1.00–1.42)	1.29 (1.05,1.60)	**<0.001**
PFT/BMI (mm/kg/m^2^)	0.44 (0.35, 0.54)	0.49 (0.41, 0.59)	0.53 (0.42, 0.67)	**<0.001**

DM-1: diabetes mellitus without albuminuria; DM-2: diabetes mellitus with microalbuminuria; DM-3: diabetes mellitus with macroalbuminuria; BMI: body mass index; BMI = weight (kg)/height^2^ (m^2^); HbA1C: glycated hemoglobin ratio; FPG: fasting plasma glucose; UTP: total 24-hour urinary protein; UCr: urinary creatinine; urine albumin: urinary microprotein; UACR: urinary creatinine protein ratio; UACR (mg/g) = urinary creatinine/urinary microalbumin; ALB: serum albumin; urea: serum urea nitrogen; SCr: blood creatinine; UA: blood uric acid; Tch: serum total cholesterol; TG: triglycerides; HDL: serum high-density lipoprotein; LDL: serum low-density lipoprotein; eGFR: predicted glomerular filtration rate; Hb: hemoglobin; PT: prothrombin time; PFT: perirenal fat thickness; OHA: oral hypoglycemic agent; Rx: prescription.

**Table 3:** The baseline characteristics of patients with L-PFT and H-PFT in the follow-up study.

	L-PFT (*n* = 41)	H-PFT (*n* = 177)	*P*
Gender (M/F)	21/20	122/55	**0.032**
Age (years)	19 (53–68)	57 (48–66)	0.140
Diabetes course (years)	4 (1–10)	5 (1–14)	0.214
Hypertension (*n*, %)	25, 61.0	102, 57.6	**<0.001**
BMI (kg/m^2^)	23.70 (20.70–28.65)	24.41 (21.52–27.37)	0.581
HbA1C (%)	9.50 (7.35–11.00)	8.30 (6.90–10.40)	0.110
FPG (mmol/L)	8.41 (6.16–12.62)	7.94 (5.99–10.88)	0.544
UACR (mg/g)	13.80 (9.53–21.48)	17.04 (12.58–21.01)	0.184
UTP (g/24 h)	0.07 ± 0.04	0.08 ± 0.02	0.561
ALB (g/L)	39.90 (37.25–42.60)	40.50 (37.65–44.15)	0.362
Urea (mmol/L)	5.86 (4.56–6.95)	5.90 (4.66–7.00)	0.401
SCr (*μ*mol/L)	70.00 (53.6–95.2)	72.00 (55.0–105.5)	0.412
UA (*μ*mol/L)	334.66 ± 112.35	338.30 ± 129.23	0.868
Tch (mmol/L)	3.35 (2.03–4.62)	3.92 (2.22–5.22)	0.103
TG (mmol/L)	2.28 (1.09–4.07)	2.53 (1.48–4.29)	0.247
HDL (mmol/L)	1.06 (0.83–1.26)	1.01 (0.85–1.28)	0.897
LDL (mmol/L)	2.27 (1.95–3.09)	2.63 (2.04–3.17)	0.338
eGFR (mL/min/1.73 m^2^)	104.40 (80.0–112.5)	105.34 (86.6–113.9)	0.630
Rx			
None (diet alone) (*n*, %)	3 (8.0)	15 (8.3)	0.285
OHA (*n*, %)	8 (19.8)	37 (21.0)	0.345
Insulin ± OHA (*n*, %)	24 (58.5)	102 (57.5)	0.114
Antihypertensive drugs (*n*, %)	15 (36.2)	63 (35.4)	0.186
ACE-I/ARBs (*n*, %)	9 (22.1)	38 (21.5)	0.201
Hypolipidemic therapy (*n*, %)	14 (34.0)	59 (33.5)	0.215
PFT (cm)	0.73 ± 0.15	1.34 ± 0.31	**<0.001**

L-PFT: lower PFT group; H-PFT: higher PFT group; BMI: body mass index; hypertension: proportion of patients with hypertension; HbA1C: glycated hemoglobin ratio; FPG: fasting plasma glucose; UTP: total 24-hour urinary protein; UACR: urinary creatinine protein ratio; ALB: serum albumin; urea: serum urea nitrogen; SCr: blood creatinine; UA: blood uric acid; Tch: total serum cholesterol; TG: triglycerides; HDL: serum high-density lipoprotein; LDL: serum low-density lipoprotein; eGFR: predicted glomerular filtration rate; PFT: perirenal fat thickness; OHA: oral hypoglycemic agent; Rx: prescription.

**Table 4:** Cox regression models for risk factors associated with the endpoint.

Model	Variable	HR	95% CI	*P*
Model 1	Sex	1.80	0.91, 3.56	0.090
	Age	1.02	0.93, 1.04	0.186
	PFT	2.91	1.16, 7.30	**0.023**

Model 2	Sex	1.53	0.72, 3.28	0.147
	Age	1.040	0.94, 1.11	0.424
	Hypertension	2.57	0.77, 4.20	0.113
	Diabetes course	1.13	0.94, 1.27	0.201
	Insulin therapy	0.48	0.25, 0.93	**0.029**
	Antihypertensive drugs	0.25	0.08, 0.83	**0.024**
	Hypolipidemic therapy	0.39	0.11, 1.11	0.152
	PFT	2.89	1.14, 7.50	**0.026**

Model 3	Sex	1.39	0.66, 2.21	0.440
	Age	1.01	0.99, 1.03	0.254
	Diabetes course	1.04	0.98, 1.05	0.511
	Hypertension	2.26	1.18, 3.38	**0.014**
	Insulin therapy	0.41	0.21, 0.67	**0.005**
	Antihypertensive drugs	1.44	0.81, 2.04	0.866
	Hypolipidemic therapy	2.11	0.41, 3.83	0.890
	PFT	2.83	1.34, 4.88	**<0.001**
	BMI	1.04	0.97, 1.11	0.288
	PFT/BMI	1.20	1.03, 1.40	**0.017**
	HbA1C	1.20	1.11, 1.26	**<0.001**
	ALB	1.03	0.98, 1.08	0.259
	eGFR	0.98	0.97, 0.99	**<0.001**
	UACR	1.06	1.01, 1.12	**0.026**

Hypertension: history of hypertension; HbA1C: glycated hemoglobin ratio; FPG: fasting plasma glucose; UACR: urinary creatinine protein ratio; ALB: serum albumin; urea: serum urea nitrogen; SCr: blood creatinine; UA: blood uric acid; Tch: total serum cholesterol; TG: triglycerides; HDL: serum high-density lipoprotein; LDL: serum low-density lipoprotein; eGFR: predicted glomerular filtration rate; PFT: perirenal fat thickness.

## Data Availability

The resources generated and/or analyzed in the present study are accessible from the corresponding authors on reasonable request.

## Supplementary Materials

Supplementary Materials Table S1: correlation between perirenal fat thickness and other statistically significant indicators. Table S2: logistic regression analysis of the presence of proteinuria in diabetic patients. Table S3: logistic regression analysis of the increase in perirenal fat thickness. Figure 1: the correlation between the L-PFT and R-PFT. Figure 2: (A) Norman diagram of the risk factors for proteinuria in patients with diabetes. Patients can calculate the corresponding points on the chart according to their UTP, age, PFT, and TG. The summed total points correspond to the risk of proteinuria. UTP: total 24-hour urinary protein; PFT: perirenal fat thickness; TG: triglycerides. (B) Norman diagram of risk factors associated with increased perirenal fat thickness. Patients can calculate the corresponding points on the chart according to their gender, UACR, and BMI, and the summed total points correspond to the risk of increased perirenal fat thickness. Sex: 1 (male) and 2 (female); ACR: 1 (ACR < 30 mg/g), 2 (30 mg ≤ UACR ≤ 300 mg/g), and 3 (UACR > 300 mg/g); BMI: body mass index.
