# Supplementary material for: The Utility of Perirenal Fat in Determining the Risk of Onset and Progression of Diabetic Kidney Disease
Source: Int J Endocrinol. 2022 Nov 30;2022:2550744. doi: 10.1155/2022/2550744 (PMC9729039; doi:10.1155/2022/2550744)
Supplement: Supplementary Materials — Table S1: correlation between perirenal fat thickness and other statistically significant indicators. Table S2: logistic regression analysis of the presence of proteinuria in diabetic patients. Table S3: logistic regression analysis of the increase in perirenal fat thickness. Figure 1: the correlation between the L-PFT and R-PFT. Figure 2: (A) Norman diagram of the risk factors for proteinuria in patients with diabetes. Patients can calculate the corresponding points on the chart according to their UTP, age, PFT, and TG. The summed total points correspond to the risk of proteinuria. UTP: total 24-hour urinary protein; PFT: perirenal fat thickness; TG: triglycerides. (B) Norman diagram of risk factors associated with increased perirenal fat thickness. Patients can calculate the corresponding points on the chart according to their gender, UACR, and BMI, and the summed total points correspond to the risk of increased perirenal fat thickness. Sex: 1 (male) and 2 (female); ACR: 1 (ACR < 30 mg/g), 2 (30 mg ≤ UACR ≤ 300 mg/g), and 3 (UACR > 300 mg/g); BMI: body mass index. [file 2550744.f1.docx]

**Supplementary**

**Table S1** Correlation between perirenal fat thickness and other statistically significant Indicators.

|  | Statistics | HbA1C | UTP | UACR | Urea | SCr | UA | TG | eGFR | BMI | hypertension |
| --- | --- | --- | --- | --- | --- | --- | --- | --- | --- | --- | --- |
| PFT | r | -0.084 | 0.177 | 0.114 | 0.089 | 0.215 | 0.159 | 0.093 | -0.142 | 0.119 | 0.137 |
|  | *p* | **0.009** | **<0.001** | **<0.001** | **0.006** | **<0.001** | **<0.001** | **0.004** | **<0.001** | **<0.001** | **<0.001** |

HbA1C: glycated hemoglobin ratio; UTP: total 24-hour protein; UACR: urinary creatinine protein ratio; Urea: serum urea nitrogen; SCr: blood creatinine; UA: blood uric acid; TG: triglycerides ; eGFR: predicted glomerular filtration rate; PFT: perirenal fat thickness; hypertension: history of hypertension or not.

**Table S2** Logistic regression analysis of the presence of proteinuria in diabetic patients.

| Univariate regression analysis | | Multivariate factor logistic regression | | | | | | |
| --- | --- | --- | --- | --- | --- | --- | --- | --- |
| Parameter | *p* | B | S.E | Wald | *p* | OR | 95%C.I.for OR | |
|  |  |  |  |  |  |  | Lower | Upper |
| UTP | **<0.001** | 5.638 | 0.670 | 70.801 | **<0.001** | 280.975 | 75.561 | 1044.816 |
| age | **<0.001** | 0.029 | 0.010 | 7.834 | **0.005** | 1.030 | 1.009 | 1.051 |
| Hb | **<0.001** | -0.001 | 0.005 | 0.014 | 0.907 | 0.999 | 0.990 | 1.009 |
| PFT | **<0.001** | 1.432 | 0.308 | 21.639 | **<0.001** | 4.186 | 2.290 | 7.653 |
| Urea | **<0.001** | -0.004 | 0.034 | 0.014 | 0.907 | 0.996 | 0.932 | 1.065 |
| SCr | **<0.001** | 0.009 | 0.005 | 3.538 | 0.060 | 1.009 | 1.000 | 1.019 |
| ALB | **<0.001** | -0.015 | 0.018 | 0.683 | 0.409 | 0.985 | 0.951 | 1.020 |
| UA | **<0.001** | 0.000 | 0.001 | 0.172 | 0.678 | 1.000 | 0.999 | 1.002 |
| TG | **<0.001** | 0.113 | 0.046 | 5.975 | **0.015** | 1.120 | 1.023 | 1.226 |
| eGFR | **<0.001** | 0.007 | 0.008 | 0.713 | 0.399 | 1.007 | 0.991 | 1.023 |
| Constant |  | -6.071 | 1.862 | 10.629 | **0.001** | 0.002 |  |  |

UTP: total 24-hour protein; Hb: hemoglobin; PFT: perirenal fat thickness; Urea: serum urea nitrogen; SCr: blood creatinine; ALB: serum albumin; UA: blood uric acid; TG: triglycerides; eGFR: predicted glomerular filtration rate.

**Table S3** Logistic regression analysis of perirenal fat thickness thickening.

| Univariate regression analysis | | Multivariate factor logistic regression | | | | | | |
| --- | --- | --- | --- | --- | --- | --- | --- | --- |
| Parameter | *p* | B | S.E | Wald | *p* | OR | 95%C.I.for OR | |
|  |  |  |  |  |  |  | Lower | Upper |
| UTP | **0.011** | 0.005 | 0.030 | 0.031 | 0.860 | 1.005 | 0.948 | 1.066 |
| sex | **0.005** | 0.318 | 0.143 | 4.939 | **0.026** | 1.374 | 1.038 | 1.818 |
| BMI | **0.003** | 0.044 | 0.017 | 6.584 | **0.010** | 1.045 | 1.010 | 1.081 |
| UACR | **<0.001** |  |  | 10.204 | **0.006** |  |  |  |
| UACR(1) | **0.015** | 0.370 | 0.169 | 4.759 | **0.029** | 1.447 | 1.038 | 2.017 |
| UACR(2) | **<0.001** | 0.707 | 0.260 | 7.416 | **0.006** | 2.028 | 1.219 | 3.373 |
| SCr | **0.005** | 0.001 | 0.002 | 0.167 | 0.683 | 1.001 | 0.997 | 1.004 |
| UA | **0.011** | 0.000 | 0.001 | 0.219 | 0.640 | 1.000 | 0.999 | 1.002 |
| TG | **0.035** | 0.023 | 0.034 | 0.454 | 0.501 | 1.023 | 0.957 | 1.094 |
| Constant |  | -1.709 | 0.451 | 14.373 | **<0.001** | 0.181 |  |  |

UTP: total 24-hour protein; sex: male; BMI: body mass index; UACR: urinary creatinine protein ratio; UACR (1): patients with 30 mg ≤ UACR ≤ 300 mg/g compared to patients with UACR < 30 mg/g; UACR (2): patients with UACR > 300 mg/g compared to patients with UACR < 30 mg/g; SCr: blood creatinine; UA: blood uric acid; TG: triglycerides.


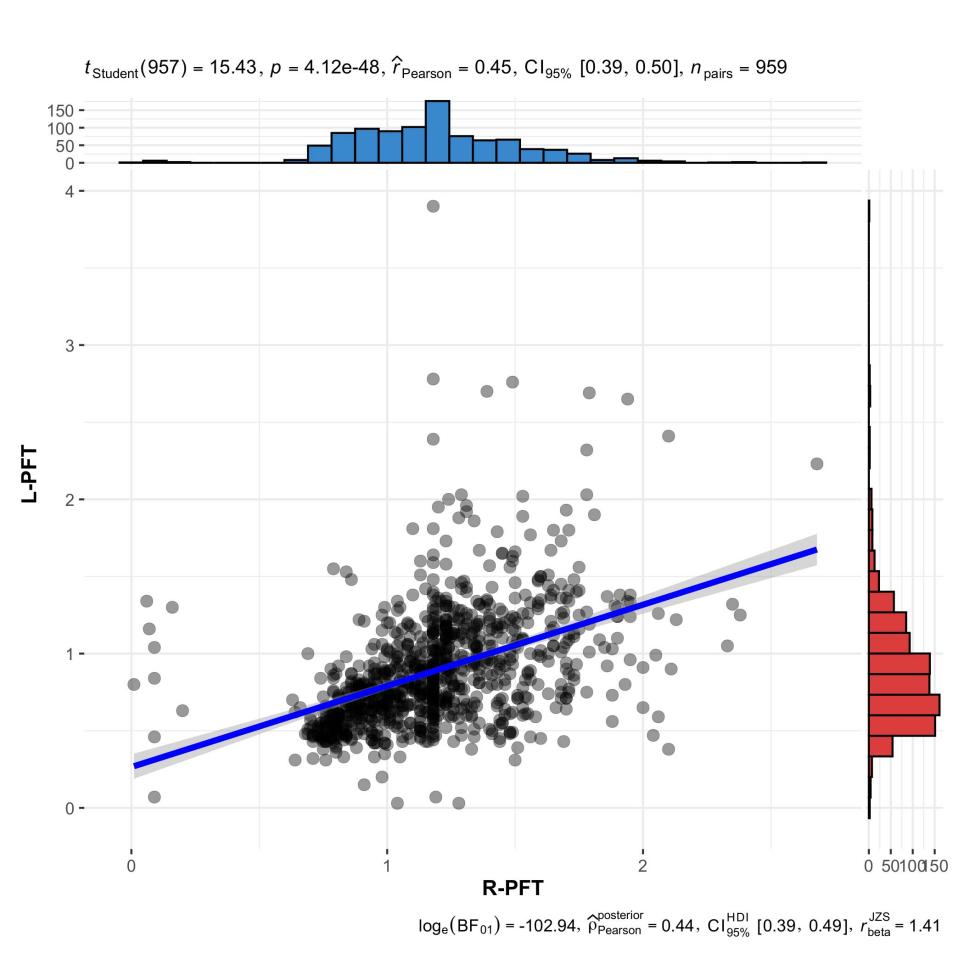


**Fig. S1. The correlation between the L-PFT and R-PFT.**


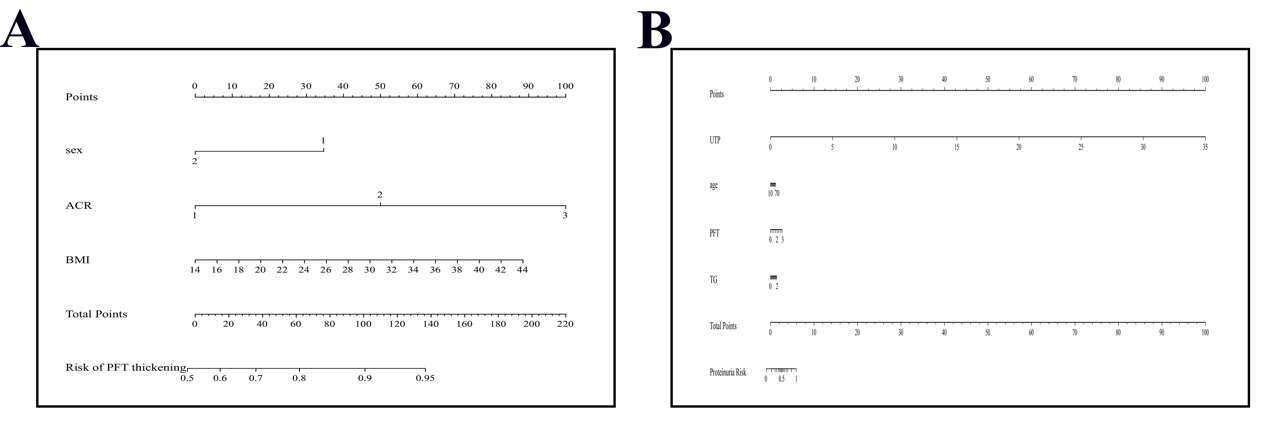


**Fig. S2.** (A) Norman diagram of risk factors for proteinuria in diabetic patients. Patients can calculate the corresponding points on the chart according to their UTP, age, PFT and TG. The summed total points correspond to the risk of proteinuria. UTP: total 24-hour urinary protein; PFT: perirenal fat thickness; TG: triglycerides. (B) Norman diagram of risk factors associated with increased perirenal fat thickness. Patients can calculate the corresponding points on the chart according to their gender, UACR and BMI, and the summed total points correspond to the risk of increased perirenal fat thickness. sex: 1 (male), 2 (female)；ACR: 1 (ACR < 30 mg/g); 2 (30 mg ≤ UACR ≤ 300 mg/g); 3 (UACR > 300 mg/g); BMI: body mass index.
